# Physical Activity and Life Satisfaction: An Empirical Study in a Population of Senior Citizens

**DOI:** 10.3389/fpsyg.2021.636914

**Published:** 2021-06-30

**Authors:** Marina Wöbbeking Sánchez, Antonio Sánchez Cabaco, Beatriz Bonete-López, José David Urchaga Litago, Manuel Joaquím Loureiro, Manuel Mejía

**Affiliations:** ^1^Department of Psychology, The Catholic University of Ávila, Ávila, Spain; ^2^Department of Psychology, The Pontifical University of Salamanca, Salamanca, Spain; ^3^Department of Health Psychology, The Miguel Hernández University of Elche, Elche, Spain; ^4^Department of Psychology and Education, University of Beira Interior, Covilhã, Portugal; ^5^Research Centre in Sports Sciences, Health Sciences and Human Development (CIDESD), Covilhã, Portugal; ^6^School of Psychology, CETYS University, Tijuana, Mexico

**Keywords:** ageing, physical activity, life satisfaction, senior citizens, healthy ageing

## Abstract

**Objective**: The specialised literature indicates that the two key aspects in active ageing are performing physical activity and life satisfaction. Regarding physical activity, this not only improves physical aspects of senior citizens, but also has a positive impact on mental well-being and satisfaction with one’s own life. The aim is to demonstrate the relationship between these two variables to explain healthy ageing.

**Method**: In a sample of 300 senior citizen subjects, the influence of various sociodemographic variables (age, sex, institutionalisation, and level of education) on the performance of physical activity and life satisfaction, is analysed. The research design is a non-experimental study with two unique cross-sectional and correlational measurement groups.

**Results:** An analysis of the results indicates that people with a higher level of education present differences in physical and motivational reserves. Furthermore, age and institutionalisation have an impact on physical reserves. Analysis using structural equation models allows key relationships between the variables analysed to be predicted, which can guide the implementation of active ageing.

**Conclusion:** Motivational reserves affect healthy cognitive ageing through their positive impact on cognitive and physical reserves.

## Introduction

Economic and sociosanitary improvements over recent decades have generated a change in sociodemographic curves on a global level, although this is more obvious in developed countries. The consequences of longevity represent a significant social achievement that poses a challenge for healthcare systems (Mari, 2018). As the World Health Organization (2016a) has pointed out, we have never lived in better conditions than we do now, but maintaining well-being levels implies holding all those involved in healthcare, including individual responsibility, responsible for developing healthy habits. This is the case, for example, for Spain, where the female population, with an average life expectancy of 85.5 years, puts it in third position worldwide, only being surpassed by their counterparts in Japan and Singapore. We, therefore, need to understand the beliefs and representations of these sectors of the population to implement programmes that have a positive impact on health behaviours (Velardi, 2018). Furthermore, we must consider that the ageing process is a stage of the life cycle that presents significant differences between individuals regarding the physical and cognitive state of senior citizens (Pérez et al., 2017), which, in some cases, leads to loss of physical, cognitive, and physical capacity (Frazão et al., 2018).

One of the relevant aspects of the way we age is the performance of physical activity, and this is seen in the literature, independently or combined with the development of other aptitudes (Diaz et al., 2019). We understand physical activity as any body movement produced by the skeletal muscles themselves that requires the expenditure of energy (World Health Organization, 2016b; Parra, 2017). But it is essential to understand the recommendations made by the World Health Organization (2010) so that people aged 65 and over manage to keep themselves in the best possible physical condition. The guidelines state that adults should perform a minimum of 150 min per week of moderate aerobic physical activity and should perform muscle strengthening activities twice a week or more. Because the performance of physical activity not only improves aesthetic aspects of senior citizens, but also has a positive impact on self-perception and mood, as well as reducing anxiety and stress (Infante et al., 2011; Windle, 2014). It is very important to highlight evidence of positive involvement in the immune system as a protective factor against disease (Guerrero et al., 2020). For this reasons, some authors (Devereux-Fitzgerald et al., 2016) have indicated the importance of interventions being focused on encouraging physical activity through fun, joyous, achievable pastimes for senior citizens, and with significant short-term benefits.

Related to physical activity is quality of life, and an important aspect, is life satisfaction, which is understood to be the key to the development of successful ageing. Positive Psychology deems well-being to be a globalising concept that includes satisfaction, happiness, life considered as a whole, high morale, personal adjustment, good attitudes towards life, and competence (Seligman, 2011). Theoretical conceptualisation, which has been proven in studies that show that active people have a better quality of life (Gomez-Piriz et al., 2014). Within this disciplinary field, the term subjective well-being, which is deemed to be a complex construct associated with one’s own experience, what is deemed to be a “good life”, and having full use of one’s faculties, is of special interest (Kahneman, 1999). Hence, two schools of thought regarding well-being emerge, the hedonic (focusing on happiness and the avoidance of pain and the pursuit of pleasure) and the eudaimonic (focusing on meaning and self-realisation), as indicated by Ryan and Deci (2001). Therefore, assessing the personal well-being of senior citizens allows us to get close to the intrinsic ageing process and understand their perceived reality. This also includes being able to understand paradoxical results such as, as the years go by, senior citizens being able to express greater satisfaction with life and a reduction in the search for meaning (Avellar et al., 2017).

Scientific literature has proven the relationship that exists between disease and low levels of well-being, as well as life satisfaction and dementia combined (Peitsch et al., 2016). Following this line, greater satisfaction has been shown in terms of the health, good functional skills, a large number of social contacts, marital status, and the educational level of the senior population (Montenegro and Soler, 2013; Martín Aranda, 2018). Over recent years, other research has shown interest in studying life satisfaction in the intermediate and final stages of life, with results that do not always coincide (Guillén and Angulo, 2016; Jiménez et al., 2016; Cabaco and Fernández, 2019).

Finally, and in relation to changes that occur in the ageing process, sociodemographic variables should be considered to assess both cognitive processes and the performance of physical activity and a person’s own life satisfaction since the importance of certain variables (age, sex, level of education, and residential care home) has already been proven as an important indicator that some functions decline to a greater or lesser extent (Arenaza-Urquijo et al., 2013; Gómez et al., 2018; Raimundo et al., 2020).

Given that, in the literature, there is still not a broad consensus on how different sociodemographic variables are related, and based on the aforementioned research, the objective of this research was derived with the intention of providing greater clarification. The relationship between gender, age, level of education, and whether or not the person is in institutional care and the performance of physical activity and the meaning of life of those aged over 55. Furthermore, we intend to establish a predictive model of the interdependence of variables associated with physical, cognitive, and motivational reserves.

## Materials and Methods

The research design is a non-experimental study with two unique cross-sectional and correlational measurement groups.

### Participants

The sample is made up of 300 subjects, 224 being women (75%) and 76 men (25%), of whom 150 subjects are in institutional care and 150 live independently. The whole sample is made up of subjects aged between 55 and 99 years with an average age for the men of 74.66 and 74.7 for the women, the overall average age being 74.68.

With respect to the group of institutionalised subjects, this is made up of 107 women (71%) and 43 men (29%), with ages ranging from 55 to 99 and whose average age is 83.17. The group of non-institutionalised subjects is made up of a total of 117 women (78%) and 33 men (22%) with ages ranging from 55 to 84 with an average age of 66.21.

Regarding the level of education of the sample, 10% have had no kind of schooling, bordering on illiteracy, 51% completed primary education, 20% secondary education, and 19% studied at university. On the other hand, the relationship between gender and education was analysed, showing that both men and women present similar levels of education throughout the sample. As can be seen, there are no difference between genders in terms of educational level.

The criteria for inclusion were the same for both groups, firstly, being 55 or older, not presenting cognitive impairment and being institutionalised in a residential care home or living at home.

### Instruments

The assessment battery consisted firstly of a sociodemographic data sheet that also includes information about the senior citizen such as gender, age, level of education, and institutional care home, in the case of those belonging to that subsample. Secondly, the battery consisted of the following questionnaires:

*International Physical Activity Questionnaire (IPAQ)*. This questionnaire was developed by the World Health Organization (2012) and is made up of seven items, whereby the replies are quantified by means of the minutes or hours spent performing an activity in the last 7 days, as indicated for each item. The reliability of the IPAQ, in its short version, is 0.65 (rs = 0.76; IC95%:0.73–0.77). With this scale, we obtain data relating to physical activity associated with health, and it is currently being used with senior citizens.
*Purpose In Life test (PIL)*. This is an attitude scale designed by Crumbaugh and Maholick (1964) to measure the degree to which an individual feels that his/her life has meaning and purpose, as well as for detecting an existential void. This test is currently the most used instrument in research on meaning of life due to high internal consistency (in all cases, greater than 0.80 in Cronbach’s Alpha) obtained in numerous studies with different populations (Martínez et al., 2012). This test is made up of 20 items, whereby the subjects should reply individually by means of a Likert-type scale from 1 to 7 between two extreme feelings.

### Procedure

Two phases were determined for the development of the research. In the first of these, the sample was recruited such that contact between the centres where the research was to be conducted was initiated, for both the independent and institutionalised groups. The first group, subjects belonging to the non-institutionalised group, is made up of students from the University of Experience of the Pontifical University of Salamanca and the SABIEX Programme of the Miguel Hernández University of Elche. Participants from the institutionalised group were obtained from various residential care homes from the Autonomous Communities of Castilla y León and Valencia, to maintain the same contextual characteristics as the first group.

Once contact had been made with each centre and institution, the second phase was launched, whereby the application of a battery of tests was undertaken. These tests were administered individually, lasting approximately an hour and a quarter for each senior citizen. The assessments were performed between the months of March and October 2017 and, in all cases, informed consent for participation was obtained beforehand. Furthermore, the study strictly complied with the ethical criteria indicated in the Helsinki Declaration (2013, review) for research of this type.

Finally, as criteria for inclusion, as well as accepting the conditions indicated (voluntary participation, presenting legal authorisation and waiving remuneration), they should not present cognitive impairment. Failure to meet any of the above criteria was grounds for exclusion.

### Analysis of the Results

For the calculation of the statistical analyses, the SPSS V.24 statistical programme was used. For the comparison between two groups, the Student *t*-test was calculated and, for the comparison between several groups, the ANOVA of a factor. Pearson’s linear correlation statistic was used to study the relationships between variables. Descriptive statistics (mean and SD) for each variable being studied were also studied.

Considering the interrelation between several variables (e.g., correlation between age and education known in Spanish population), a structural analysis was performed using the lavaan package (Rosseel, 2012) in R statistical language. The tested model included all four predictors (age, education, sex, and institutionalisation) and both dependent variables (life satisfaction and physical activity) simultaneously. Common fit indices were calculated: root mean square error of approximation (RMSEA), comparative fit index (CFI), standardised root mean residual (SRMR), and Tucker-Lewis index (TLI). Indications of a good fit were values of 0.08 or lower for the RMSEA and SRMR, and values of 0.90 or higher for CFI and TLI. Akaike Information Criterion (AIC) and Bayesian Information Criterion (BIC) were used to confirm if the model with all predictors included (sex, education, age, and institutionalisation) showed a better fit than simpler models: one with only age as a predictor, and another one only modelling the means of physical activity and life satisfaction. Smaller AIC and BIC values indicate better fit.

## Results

Regarding the first objective, the results found for the sociodemographic variables analysed are shown. For all these, physical reserves (assessed using the IPAQ) and motivational (assessed using meaning of life with the PIL test) measurements are considered. On both these scales, the scores are interpreted such that the higher the score in the PIL test, the greater the meaning of life (motivational reserves); and, on the IPAQ scale, the higher the score, the less activity and, therefore, lower physical reserves.

With respect to possible differences according to *gender*, after performing contrast analysis using the Student *t*-test, no significant differences were obtained between men and women (*p* > 0.05), or in physical or motivational reserves (see Table 1).

**Table 1:** Differences by sex in physical and motivational reserves.

	Sex (mean, SD^1^)		Levene test (sig.)	*t*-test (sig.)
	Men	Women		
Motivational reserves (PIL)	140.5 (19.1)	140.9 (17.2)	0.177	0.865
Physical reserves (IPAQ)	2.07 (0.783)	1.97 (0.765)	0.423	0.345

1 SD, standard deviation.

As regards the relationship between *age* and both physical and motivational reserves, Pearson’s linear correlation test shows that there is no significant correlation between age and meaning of life (r_xy_: 0.050; sig: 0.351 > 0.05); although there is between age and physical activity (IPAQ), indicating that, the older they are, the less physical activity senior citizens do (r_xy_: 0.196; sig: 0.001 < 0.05).

The third sociodemographic variable refers to *educational level* to determine whether there are differences in physical and motivational reserves. For Meaning of Life (PIL), means contrast only shows significant differences (*p* < 0.05) between the “Without Studies” (SE) and “University” (UN) groups, such that the university educated group scores higher (greater meaning of life; see Table 2).

**Table 2:** Differences according to educational level and physical and motivational reserves.

Reserves		Educational level				Contrast tests		
		SE^1^	PR^1^	ES^1^	UN^1^	PL^2^	PA^2^	Comparison between groups (P. Scheffe)
PIL	Mean	133.9	141.0	138.3	145.7	0.837	0.026	SE = PR, ES; SE < UN^*^
	SD	18.76	18.90	16.38	18.85			PR = ES, UN
	N	30	152	60	58			ES = UN
IPAQ	Mean	2.60	2.19	1.68	1.76	0.129	<0.001	SE < PR^*^; SE < ES, UN^**^
	SD	0.498	0.761	0.725	0.733			PR < ES, UN^**^
	N	30	152	60	58			ES = UN

1 SE, without studies; PR, primary studies; ES, secondary studies; UN, university.

2 PL: Levene’s test (significance); PA: ANOVA test (significance).

* Significance: <0.05.

** Significance: <0.001.

With respect to Physical Activity (IPAQ), it can be seen that the trend is that more physical activity is performed as the level of education rises, such that, the without studies group performs less physical activity than the primary studies group, and these do less than the secondary or university education groups (there are no differences between secondary and university education; see Table 2).

Finally, we studied whether there are differences between senior citizens regarding physical activity and meaning of life, according to whether they live in an *institutionalised environment* (care home) or not (at home). Mean contrast (*t*-test) shows that there are no significant differences for meaning of life, although there were for physical activity (sig <0.001; TE: 0.59), indicating that the non-institutionalised group perform more physical activity than the institutionalised (see Table 3).

**Table 3:** Differences according to life context (institutionalised or not) on physical and motivational reserves.

	Institutionalised (mean, SD^1^)		Levene test (sig.)	*t*-test (sig.)	TE^2^ Cohen’s *D*
	No	Yes			
Motivational reserves (PIL)	141.4 (18.2)	139.9 (18.9)	0.610	0.515	0.11
Physical reserves (IPAQ)	1.83 (0.76)	2.27 (0.74)	0.891	<0.001	0.59

1 SD, standard deviation.

2 TE, effect size (Cohen’s *D* test).

Finally, in order to separate out the effects that each assessed variable has, and to integrate it into the other variables, in other words, to properly monitor the separate effects, a structural analysis was performed that would allow the effects to be broken down.

A model with the variables Institutionalisation (whether the senior citizen is institutionalised, 0 = No, 1 = Yes), Gender (0 = Male, 1 = Female), Education (an ordinal variable, 1 = Without studies, 2 = Primary, 3 = Secondary, 4 = University), and Age as predictors, and both variables of physical activity (IPAQ) and life satisfaction (PIL) as dependent variables. The model showed an excellent fit, as the main indices show, CFI = 1, TLI = 1, RMSEA = 0, SRMR = 0. Comparison indices between models show that this model has a better fit (AIC = 3,264, BIC = 3,312) than one in which the only predictor is age (AIC = 3,288, BIC = 3,314), and another one only modelling the means for physical activity and life satisfaction (AIC = 3,299, BIC = 3,317).

**Figure 1:**
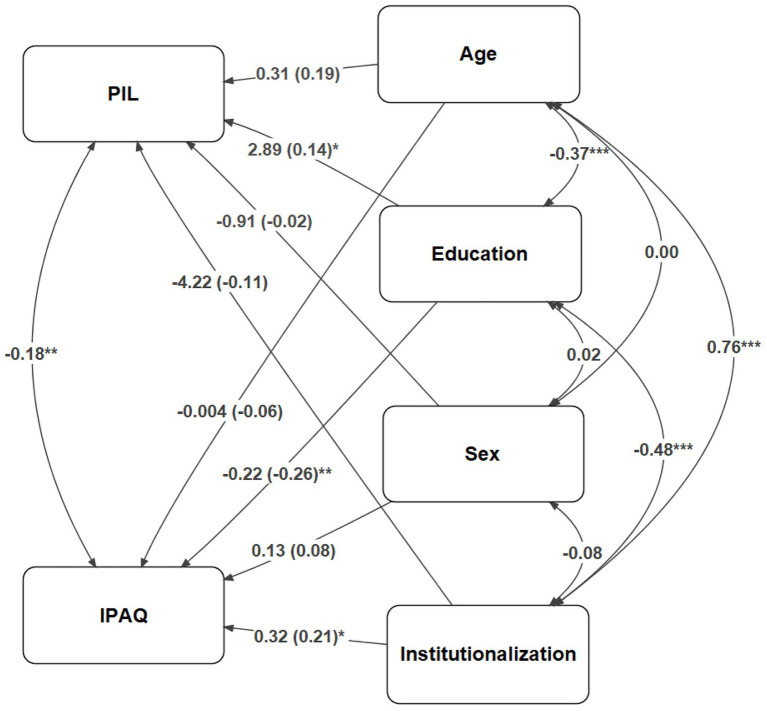
Structural equations model with the sociodemographic variables institutionalisation, gender (sex), education, and age as predictors of physical activity (IPAQ) and life satisfaction (PIL). Institutionalisation: 0 = Non-institutionalised, 1 = Institutionalised. Sex: 0 = Male, 1 = Female. Education: 1 = Without Studies, 2 = Primary, 3 = Secondary, 4 = University. After taking into account the correlations between predictors, institutionalisation remains a predictor of physical activity, and education remains a predictor of both physical activity and life satisfaction. Values for one-sided arrows: natural estimates (standardised values). Values for two-sided arrows: standardised correlation indices. ^*^p < 0.05; ^**^p < 0.01; ^***^p < 0.001.

Figure 1 shows the results whereby it can be seen that, by controlling the effects of all the variables at the same time, educational level remains an important predictor of both physical and motivational reserves. The higher the educational level, the more physical activity is performed, and the greater the life satisfaction.

It can also be seen that the factor of being institutionalised reduces physical activity. However, age is no longer a relevant factor for physical activity. This would be explained by the age differences between non-institutionalised and institutionalised subjects, since age was higher in the latter group (66.2 vs. 83.2 years old). Finally, there is a statistically significant correlation between physical activity and life satisfaction (−0.18, *p* < 0.01).

These analyses strengthen the conclusion that educational level and institutionalisation have separate effects on physical activity and life satisfaction, as well as a correlation between them that is added to these sociodemographic indicators.

## Discussion

In light of the results obtained, we can affirm that, with respect to the role sociodemographic variables play in physical and motivational reserves, there are no differences between sexes; by contrast, those with higher educational levels do present greater physical and motivational reserves.

Although in this study there are no differences between men and women, it is important to highlight the predominance of the female sex in old age, since life expectancy at birth is 85.4 years compared to that of the male sex who is in 79.9 years (Abellán et al., 2017). This can be understood due to the fact that in the programmes for the elderly, there are more women than men, as is the case in general with the elderly population (Moreno-Crespo et al., 2018; INE, 2020, 2021). In relation to the age difference in the groups of institutionalised subjects in residential centers and the group of non-institutionalised subjects, it is due to the fact that in Spain, as indicated by the INE (2020), there is a population of 46,934,632 people, corresponding to 19.64% to people over 64 years of age. In addition, regarding life expectancy, the Spanish population lives an average of 83.5 years, which is much higher than the rest of the world.

The Continuous Household Survey published by the INE (2020), states that 2,037,700 people aged 65 or over live alone in Spain (43.1%), of which 1,465,600 (71%) are women and 270,000 older people live in residential centers (Barrera-Algarín et al., 2021).

Regarding the education variable, it is observed that in the group of institutionalised subjects, the educational level is lower, because the mean age is higher, as stated by Estrada et al. (2013) in a study carried out with 276 institutionalised older adults, in which the predominant educational level was primary (51.1%), followed by secondary (25%), it was found that 17.4% did not have any level of training.

With regard to the other variables, age relates to physical reserves (the older they are, the less physical activity they perform) but not to motivational reserves, as is the institutionalisation variable (non-institutionalised people perform more physical activity). The results broadly confirm those found in the initial literature (Martín Aranda, 2018; Guerrero et al., 2020). However, there are studies that demonstrate the existence of a significant disparity in the prevalence of physical inactivity. According to the World Health Organization (2015), this prevalence is higher among women than men, which differs from the results obtained in this study. By contrast, and regarding age, it indicates that senior citizens are less active than young people. Along the same line, Crombie et al. (2009) have shown that there is a reduction in physical activity among the adult population.

A recent study carried out with 415 non-institutionalised older women described the influence of age on the level of physical activity, muscle mass, and strength in older women. In this sample, the amount of muscle mass showed an association with upper and lower limb strength and physical performance. Therefore, they concluded that the increase in age, muscle mass, and strength gradually decreased (Enríquez et al., 2019).

Regarding life satisfaction, the studies indicate that there are two related concepts, which are the feeling of presence and the search for meaning. Avellar et al. (2017) have assessed the extent to which the feeling of presence and the search for meaning change according to life cycle. They conclude that as life stages advance, meaning increases, although, by contrast, search for meaning decreases. On the other hand, their results indicated that those included in the senior citizens group perceived greater presence of meaning in the present compared to the group of young people. Although, in our study, we do not have a young sample for comparison, the results are similar to other research developed using the same instrument, even with clinical populations (Armas et al., 2018).

In a study carried out by Chacón et al. (2017), they related the performance of physical activity with a person’s state of mind. This research was conducted with a total of 1,002 subjects over the age of 18 and subdivided into age groups by means of a descriptive and correlational non-experimental study. The instruments used were, to measure physical activity they used the questionnaire designed by the IKERKI Group (Arribas et al., 2006), whereby those people who have performed some kind of physical-sporting activity over the last 12 months are deemed to perform physical activity, and, secondly, the State of Mind Questionnaire (CEA; Arruza et al., 2008), which measures the degree of presence of positive and negative feelings over the last year. The results indicated that those people who perform any kind of physical activity have a more positive state of mind than those who have not performed any kind of activity as these are characterised by a greater presence of sadness, tiredness, etc.

Carmona (2009) carried out a study that aimed to analyse personal well-being and factors that facilitate its development and maintenance. The results showed that there is a significant predictive relationship between education, health, independence, and social interactions and the personal well-being of senior citizens. By contrast, the age, marital status, sex, and socioeconomic variables did not contribute in a significant way to the personal well-being of participants in the study, results that coincide with those obtained in this research. Along this line, authors such as Montenegro and Soler (2013) have affirmed that satisfaction increases according to the health, good functional capacities, a larger number of social contacts, marital status, and educational level of senior citizens.

In another study, Franke, 2013 carried out with senior citizens in which the PIL questionnaire was used, it was observed that more elderly people presented significantly more meaning of life. This line is still in an embryonic stage with the development of the gerotranscendence construct (Tornstam, 2011), and there is a debate (ethics of care vs. ethics of needs) about which are the keys to be included in both diagnostic research as well as derived lines of intervention (Cabaco and Fernández, 2019).

Other studies (Vinaccia et al., 2017) affirm the importance of assessing the relationship between quality of life and health and perception of disease. They deem it to be important to study these variables since, according to Palomera (2009), happiness consists of positive and negative emotions as well as life satisfaction, indicating that, if the person presenting an illness maintains a positive emotional state, this results in an improvement in his/her well-being. According to Quiceno and Vinaccia (2010), cognitive and emotional factors are key in the patient’s perception of his/her illness, which has an impact on his/her progress.

García et al. (2017) have studied, using a descriptive correlational design (in a sample of 140 retired people, 90 women and 50 men with an average age of 61.23 years), the relationship between sociodemographic variables and the quality of life of these participants. The results affirm that there is a significant correlation between perception of quality of life and variables such as retirement age and physical activity. Continuing with the results, the relationship between sociodemographic variables and dimensions of quality of life, they identified significant correlations between the age of the participant and dimensions such as physical health, psychology, and social relationships. Variables that have been studied in other research (Rojas, 2011), that show that the higher the age of retirement, the lower the perception of the quality of life in these areas. Similar results were found by Tanferri et al. (2017) whereby differences appear between these dimensions and retirement age. Results that suggest that the greater the age, the lower the quality of the dimensions of physical health, psychology, and social relationships. Although the important modulating role played by cognitive reserves in mitigating cognitive impairment should be considered (Wöbbeking et al., 2020).

This study shows that motivational reserves (assessed in this study using meaning of life) have a healthy impact on physical reserves. This evidence and already considering the age, sex, educational level, and institutionalisation status of senior citizens.

Bearing all the variables of this study in mind, it seems obvious that, in light of the literature reviewed, that facing the challenges of demographic changes inevitably involves the strengthening of dimensions associated with the CR construct as a driver of healthy ageing. Particularities deriving from the circumstances of senior citizens (institutionalisation), or other keys of an ideographic or personal nature, should be the object of further research.

As far as the continuity of this line of research is concerned, in order to overcome some of the limitations of the work described, it would be necessary to be able to carry out the study in a longitudinal way, broaden the sociodemographic variables (especially those linked to cognitive reserves) and delve more deeply into motivations that influence the performance of physical activity. It will also be relevant to identify the relationship between age, institutionalisation, and physical activity with larger samples and controlling the years spent in care homes factor. The reason for the proposal is justified in that the results presented herein indicate that institutionalised senior citizens could possibly benefit from spending more time performing physical activity, adjusted to their capacities, as part of the activities of their geriatric centre.

An interesting point, which is revealed by the correlation found between physical activity and life satisfaction, and which goes beyond what is explained by the sociodemographic variables reviewed, is in the possibility of influencing life satisfaction through physical activity, or vice versa.

## Data Availability Statement

The original contributions presented in the study are included in the article/supplementary material, further inquiries can be directed to the corresponding author.

## Ethics Statement

Ethical review and approval was not required for the study on human participants in accordance with the local legislation and institutional requirements. The patients/participants provided their written informed consent to participate in this study.

## Author Contributions

MW, AS, and BB-L contributed to the development and design of the study, as well as to the application and correction of tests. JDU and MM performed the statistical analyses and wrote the methodology section of the manuscript. ML carried out a full review of the article, including bibliographical references. All authors contributed to the article and approved the submitted version.

### Conflict of Interest

The authors declare that the research was conducted in the absence of any commercial or financial relationships that could be construed as a potential conflict of interest.
